# Supplementary material for: Parallel factor ChIP provides essential internal control for quantitative differential ChIP-seq
Source: Nucleic Acids Res. 2018 Apr 17;46(12):e75. doi: 10.1093/nar/gky252 (PMC6093181; doi:10.1093/nar/gky252)
Supplement: Supplementary Data [file gky252_supplemental_files.pdf]

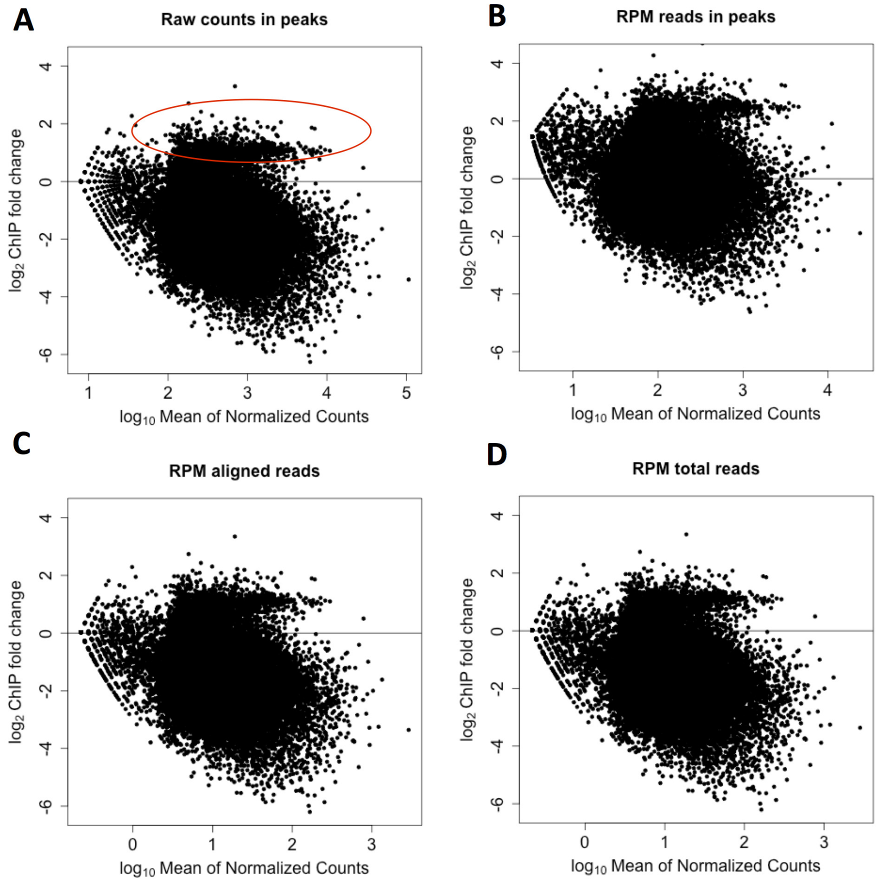

Figure S1: **Comparison of simple normalisation strategies employed.** MA plots showing the changes in ER binding after 48 hours treatment with 100 nM fulvestrant. Three simple normalisation methods were applied to this data and compared to the raw count data: (A) Raw counts, (B) Reads Per Million (RPM) reads in peaks, (C) RPM aligned reads, and (D) RPM total reads. Note that the highlighted peaks remain above zero under all three standard normalisations.

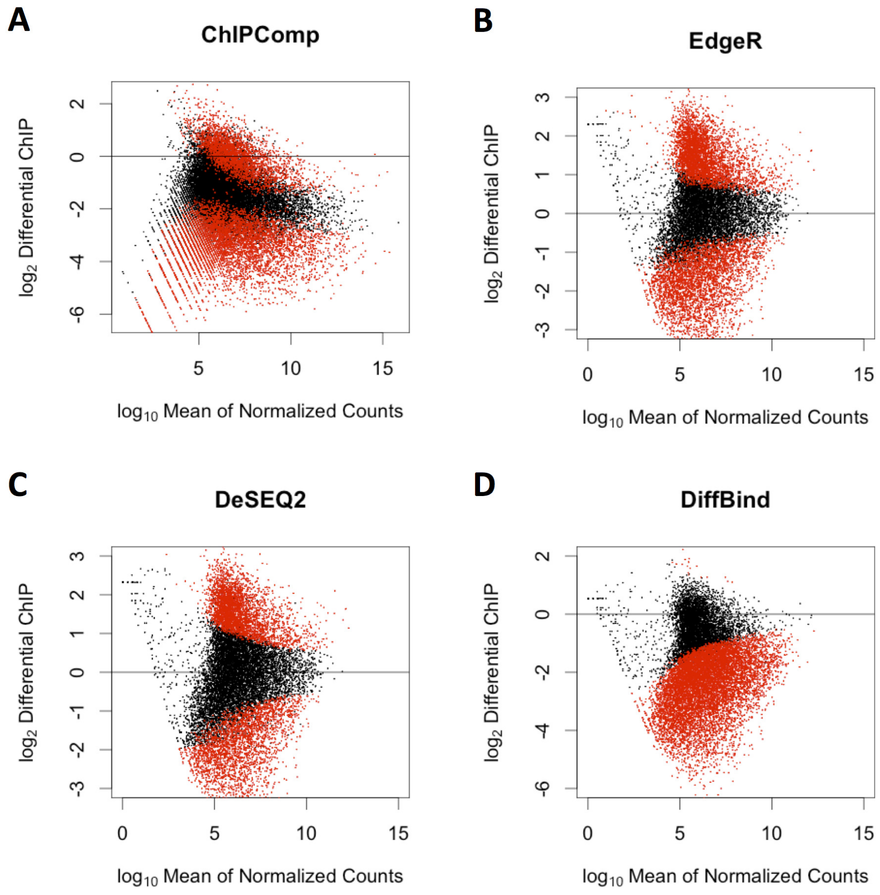

Figure S2: **Comparison of ChIP-seq Pipelines.** (A) ChIPComp data was plotted from the *CountSet* object; results show a high number of false positive upregulated sites. (B) EdgeR normalisation is designed for the analysis of transcriptional data. In the case of large-scale unidirectional changes in binding, the assumption of normalisation fails giving rise to a distribution that is artificially symmetric.(C) DeSEQ2 makes use of similar assumptions and results in a similar distortion of data. (D) DiffBind utilises normalisation to total library size, and performs significantly better than the other three methods but does not attempt to control for systematic bias in pull-down efficiency of the ChIP.

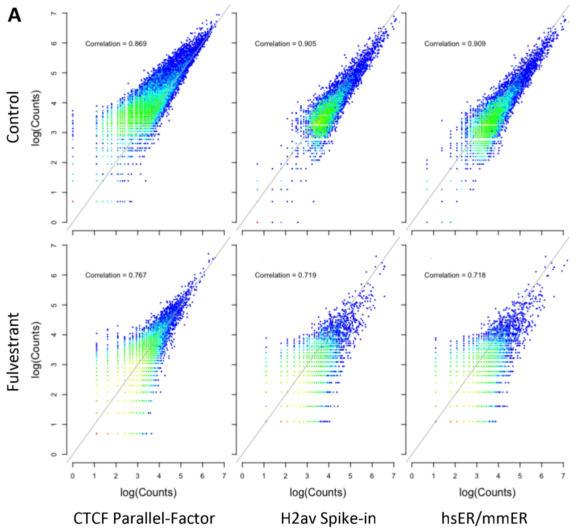

B

CTCF Parallel-Factor

FULVESTRANT \ CONTROL	Rep1	Rep2	Rep3
Rep1		0.884	0.96
Rep2	0.778		0.869
Rep3	0.767	0.855	

H2av Spike-in

FULVESTRANT \ CONTROL	Rep1	Rep2	Rep3	Rep4
Rep1		0.935	0.925	0.922
Rep2	0.833		0.938	0.933
Rep3	0.806	0.88		0.905
Rep4	0.719	0.801	0.8	

hsER/mmER

FULVESTRANT \ CONTROL	Rep1	Rep2	Rep3	Rep4
Rep1		0.932	0.923	0.922
Rep2	0.83		0.939	0.937
Rep3	0.803	0.881		0.909
Rep4	0.718	0.797	0.799	

Figure S3: **Correlation Plots of Replicate Experiments.** (A) Scatter plots showing the correlation between the replicates with the lowest correlation value. This is provided for both the control (top) and treatment (bottom) conditions. The plotted condition is highlighted with thick borders in tables on the right. Colour represents density: blue = lowest, red = highest. (B) Tables showing the correlation coefficient for each replicate. Table is divided in two; values in the bottom left are between fulvestrant replicates, values in the top right are between control replicates.

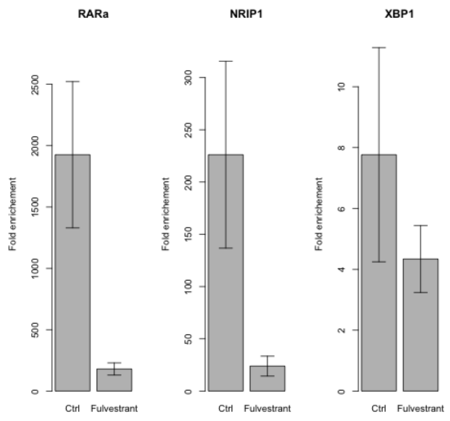

Figure S4: **ChIP-qPCR validation of ChIP data.** Loss of ER binding at RARα, NRIP1, and XBP1 enhancers was monitored by qPCR. All three sites show a reduction in binding at 48 hours after treatment with 100 nM Fulvestrant. Error bars show standard error.

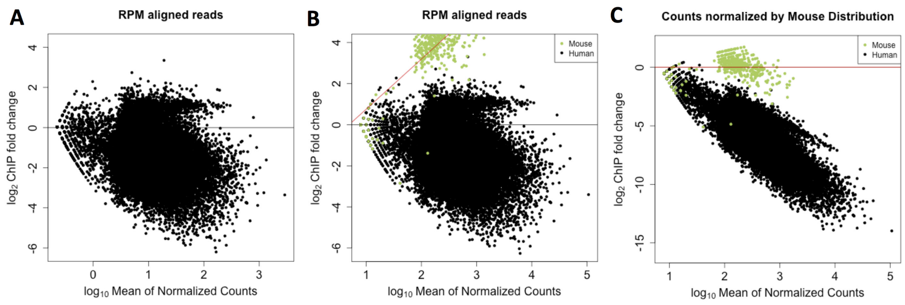

Figure S5: **MA plots showing the addition of Mm derived chromatin spike-in to the ChIP-seq analysis of MCF-7 before and after treatment with fulvestrant.** (A) MA plot after scaling factor based normalisation shows same characteristic grouping of peaks off axis. (B) ER binding in Mm samples shows considerable increase in binding after treatment of the MCF-7 cell line with fulvestrant. (C) Attempting to fit a correction factor to the data results in a significant distortion.

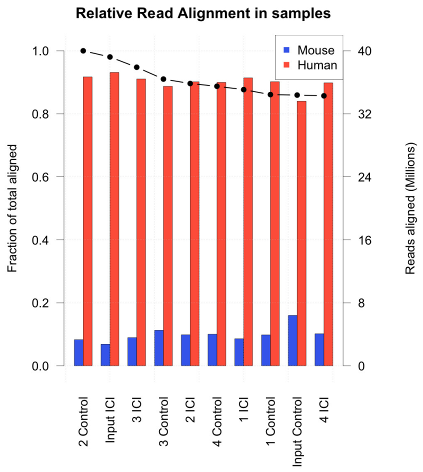

Figure S6: **Distribution of reads for Mm chromatin spike-in normalisation strategy.** Comparison of murine chromatin between samples showed no systematic bias in the sample preparation. Bar plots (left axis) represent the fraction of total aligned reads. The dot plot represents the total aligned reads (right axis) for each sample.

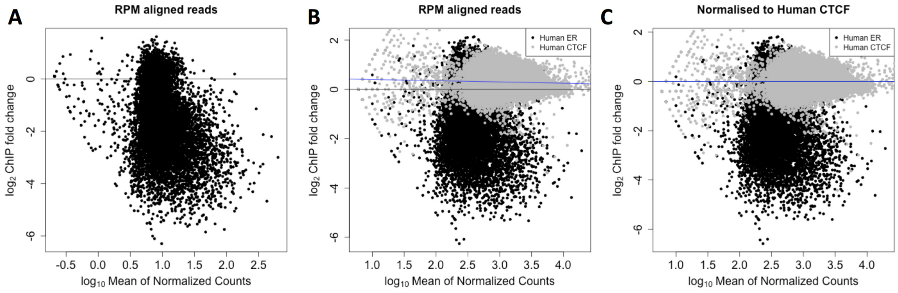

Figure S7: **MA plots showing ER binding before and after treatment with fulvestrant including matched CTCF control.** (A) Reads corrected to total aligned reads showed the same off-centre peak density as observed with all that was not-normalised with an internal spike-in control. (B) Overlaying the MA plot combining the changes in chromatin binding of ER (black) and CTCF (grey). CTCF peaks overlay the off-centre peak density. (C) Utilising the CTCF binding events as a ground truth for 0-fold change, a linear fit to the log-fold change is generated (blue line). The fit is then also applied to the ER binding events.

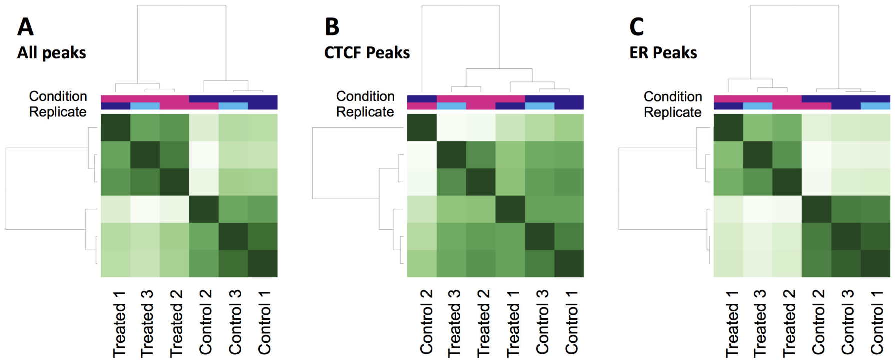

Figure S8: **Clustering of samples before and after ER and CTCF peak extractions shows the effect of fulvestrant on ER peaks drive clustering of the raw data.** To confirm that the effects seen in Figure 2 were consistent across the genome, we compared the clustering of the CTCF and the ER peaks with respect to the treatment with fulvestrant. Initial clustering was weakly correlated with that of the treatment condition (Figure S8A). Clustering specifically to CTCF derived peak data (Figure S8B) resulted in a loss of grouping by treatment, while clustering specifically ER-derived peak data (Figure S8C) led to a clearer separation by treatment.

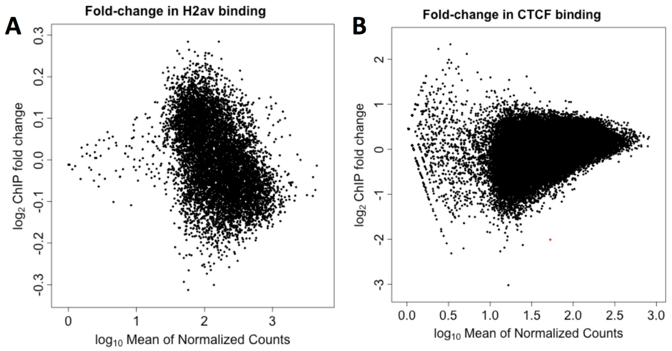

Figure S9: **Comparison of the control regions used to normalise ER analysis before and after treatment.** Dots highlighted in red are significant (FDR = 0.01). (A) H2Av occupancy of the Drosophila genome shows no significant changes before and after treatment. (B) The CTCF peaks used for normalisation show no significant change in the number reads before and after treatment.

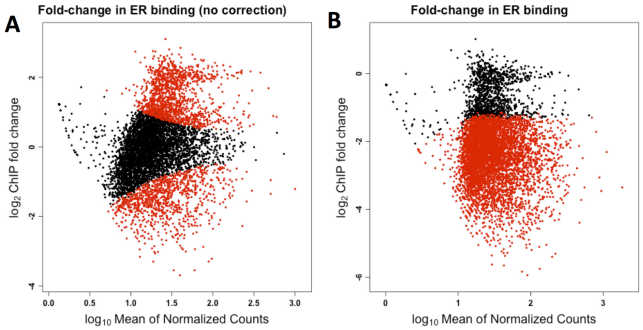

Figure S10: **Normalisation of ER binding external spike implemented using DESeq2.** Highlighted data points are considered significant fold-changes with a FDR = 0.01. (A) Initial analysis of the ER binding with default parameters shows an equal increase and decrease in ER binding. The distribution seen is not reflective of the documented response of ER on treatment with fulvestrant. (B) Estimating the DESeq2 size factors from the sample spike-in corrects the distortion in the results.

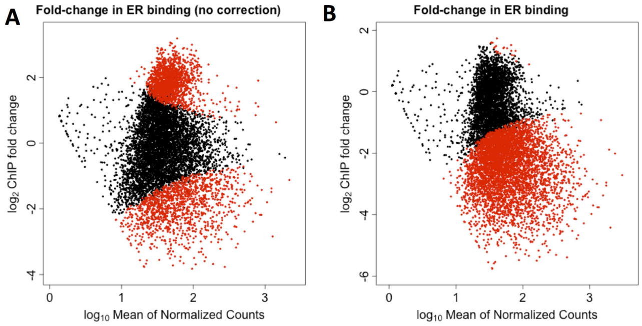

Figure S11: **Normalisation of ER binding internal CTCF control.** Highlighted data points are considered significant fold-changes with a FDR = 0.01. (A) Initial analysis with default DESeq2 parameters gives similar distortion as seen in Figure S10A. (B) Correction using the CTCF peaks to provide an internal control allows for the data to be corrected.

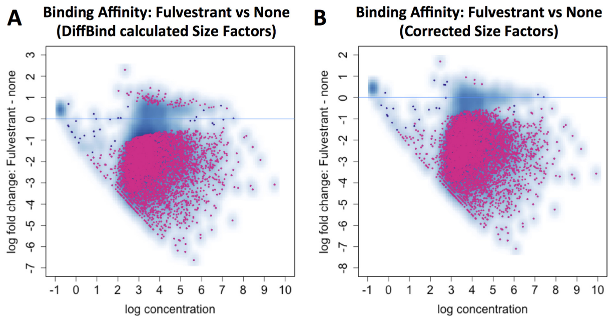

Figure S12: **Comparison of DiffBind output before and after applying the corrected size factors from our pipeline generated from Drosophila spike-in control.** (A) Analysis of ER binding before and after treatment with fulvestrant demonstrates that DiffBinds default normalisation strategy is more effective than the DESeq2 default (Fig S10A), but demonstrates a bias between samples. (B) Applying the correct size factors from our DESeq2 pipeline reduces the bias in the analysis.

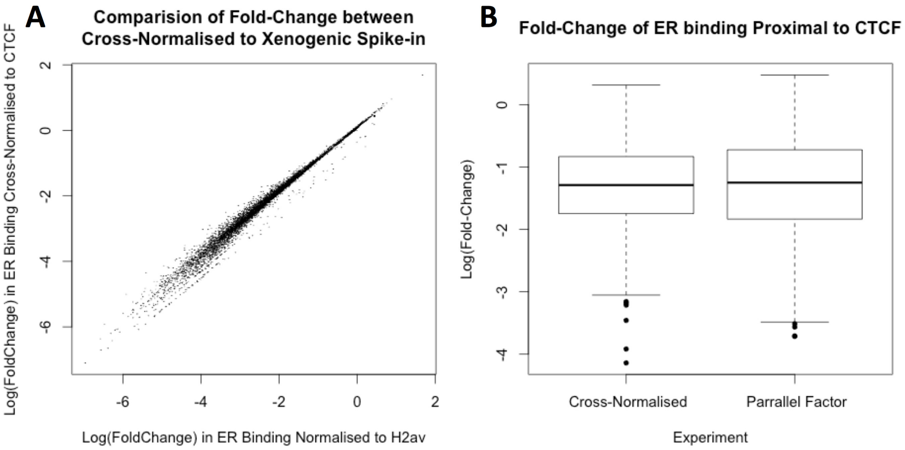

Figure S13: **Comparison of fold-change of ER binding after both xenogeneic and cross-normalisation.** (A) Scatter plot of fold-change as established at individual sites by each method. Pearson's correlation between the two methods is 0.992 (p-value tending to 0). Deviation of data points from parity is a result of the integer nature of read counts; nonetheless, the effect is very small as demonstrated by the correlation coefficient between the two datasets. (B) Box-plot showing the fold-change of ER binding before and after treatment at ∼550 ER sites proximal to CTCF binding. The mean and maximum fold-change is reduced at these sites by Parallel-Factor ChiP, but the effect is marginal.

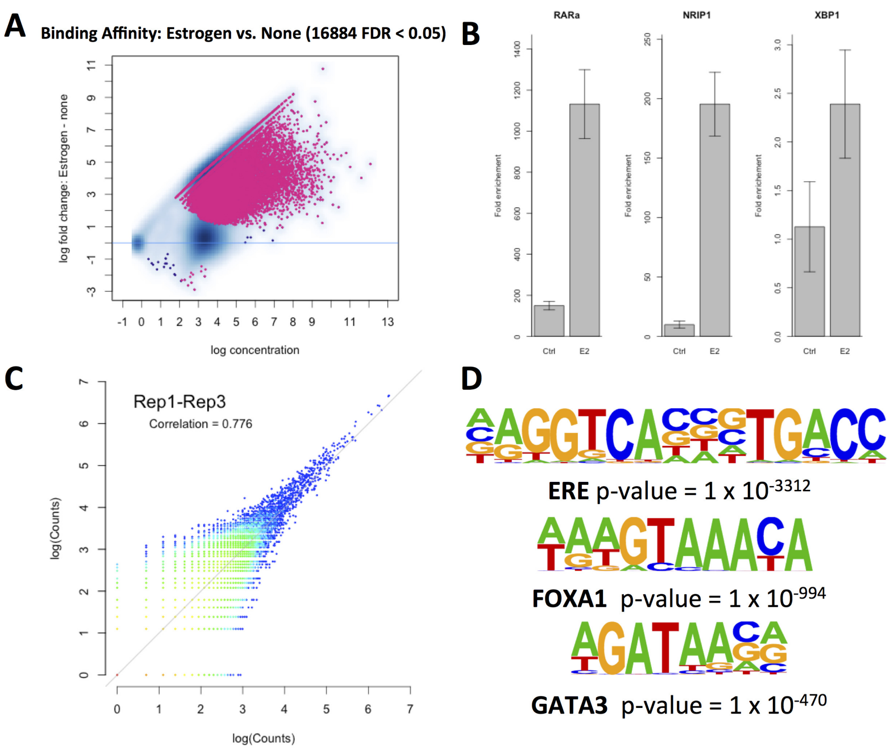

Figure S14: **Comparison of fold-change of ER binding before and after treatment with estradiol.** (A) MA plot of ER binding after normalisation to CTCF binding displays a significant increase in ER binding at 45 minutes after treatment with estradiol. (B) Binding at known ER sites proximal to RARα, NRIP1 and XBP1 all show an increase in binding 45 minutes after 100nM E2 treatment. (C) Comparison of log(Counts) for binding sites was undertaken to confirm reproducibility. The data with the lowest correlation is shown and was seen between Replicate 1 and Replicate 3 in the control condition. (D) Motif analysis of sites with significantly increased binding found motifs of the ERE, FOXA1 and GATA3 core parts of the ER complex enriched.

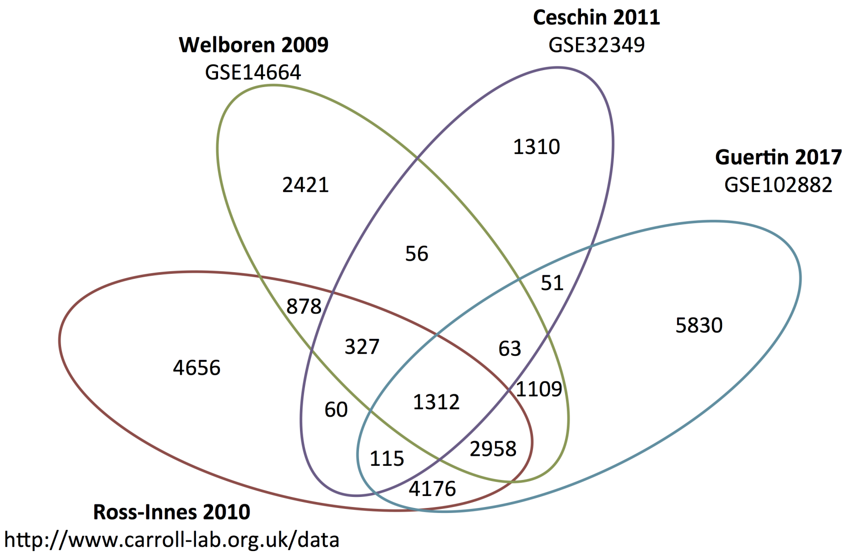

Figure S15: **Comparison ER binding from public datasets.**Common peaks detected for ER Ross-Innes CS, et al. 2010; Welboren WJ, et al., 2009; Ceschin DG, et al. 2011 and our data (FDR = 0.01). Venn diagram was generated with ChIPSeqAnno.

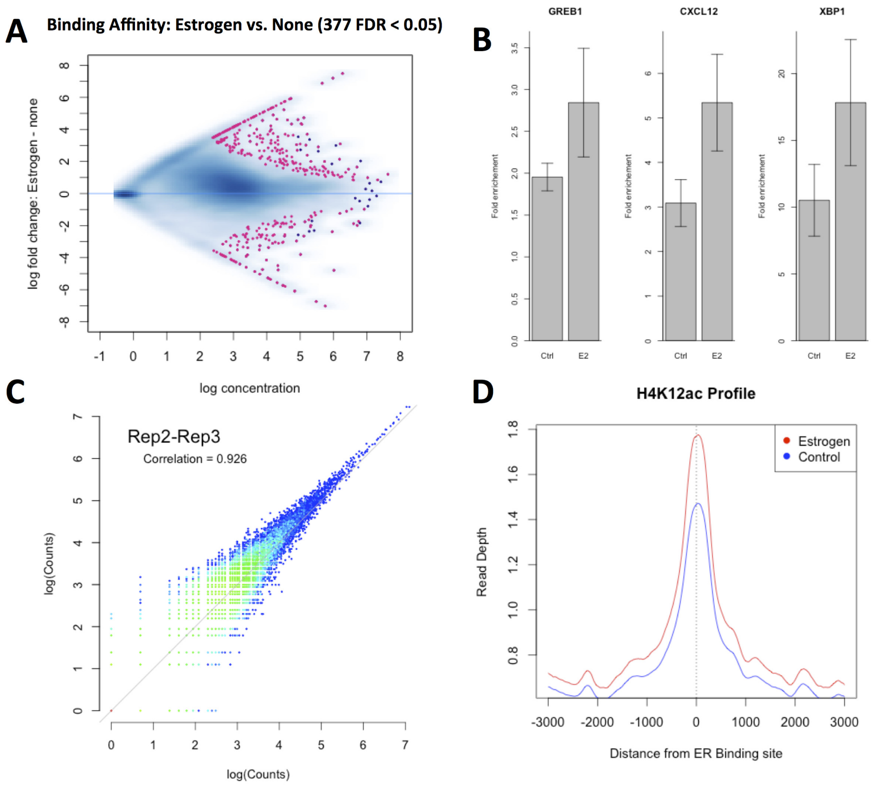

Figure S16: **Comparison of fold-change of H4 acetylation (Lys12) before and after treatment with estradiol.** (A) MA plot of H4K12ac after normalisation to CTCF binding displays an increase at 2 hours after treatment with estradiol. (B) H4K12ac occupancy proximal to known ER sites 2 hours after 100nM E2 treatment. (C) Comparison of log(Counts) for binding sites was under taken to confirm reproducibility. The data with the lowest correlation is shown and was seen between Replicate 2 and Replicate 3 in the control condition. (D) H4K12ac occupancy profile before and after treatment with E2 shows a general increase around ER binding sites.

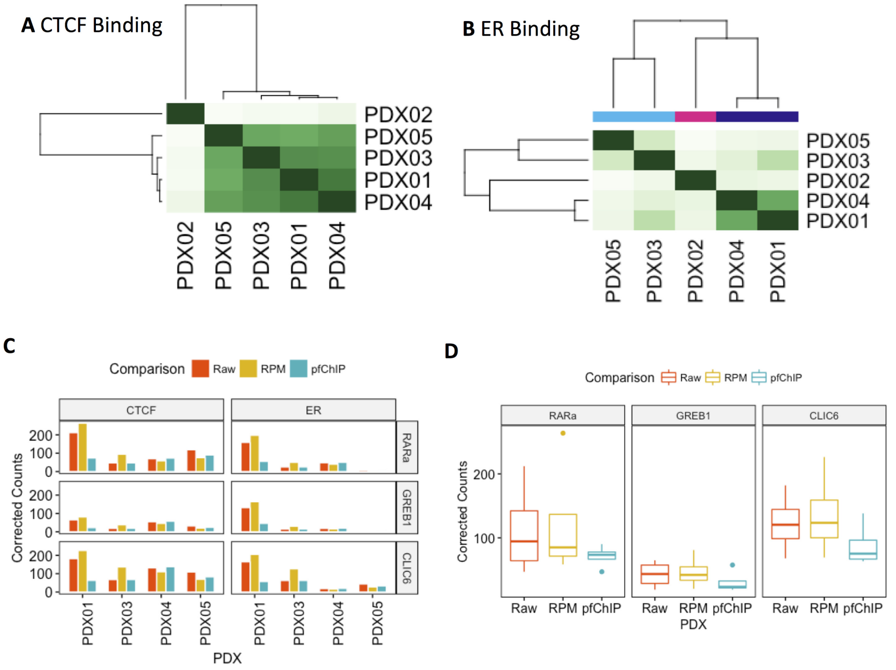

Figure S17: **Parallel-Factor ChIP-seq applied to PDX material.** (A) Correlation heatmap generated from CTCF binding patterns shows that all samples except PDX02 are highly correlated. Darker green represents greater correlation, therefore this visulisation provides a QC step, identifying the ChIP of PDX02 has failed. (B) Heatmap generated from the correlation of ER binding patterns shows a much greater distance between PDX samples compared to CTCF, implying that different tumour models have ER binding distributed differently around the genome. PDX01 and PDX04 cluster most closely; a potential reason is PDX01 and PDX04 both express PR while PDX05 is classified as PR negative. The status of PDX03 is unknown.(C) Counts in peaks near three key loci normalise differently depending if RPM or parallel-factor ChIP (pfChIP-seq) is used. (D) CTCF control peaks show a reduced variability at each locus after normalisation with parallel-factor ChIP. RPM has minimal effect, demonstrating that parallel-ChIP is more able to control for sample variability.

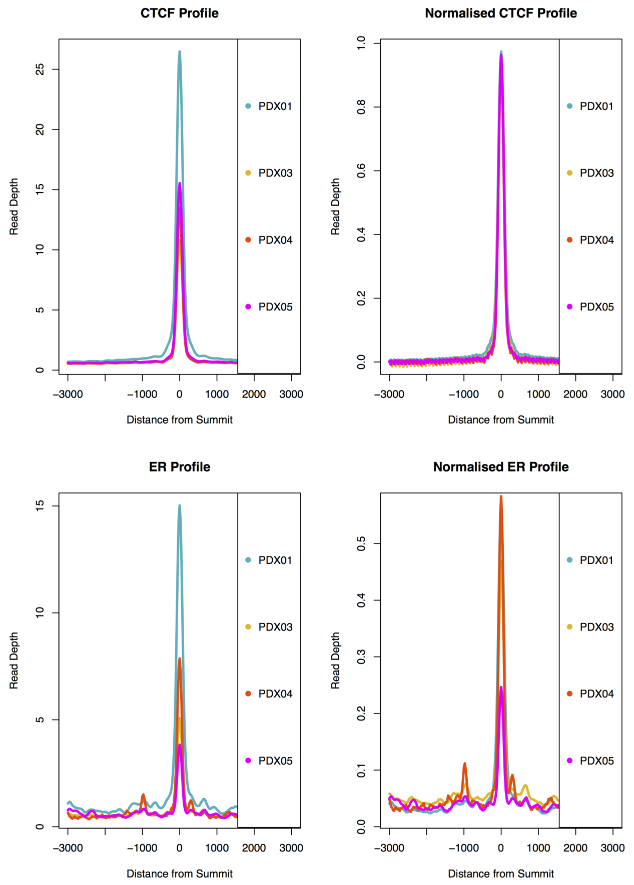

Figure S18: **Genome wide profile of parallel-factor ChIP-seq applied to PDX material.** Analysis of CTCF binding of the raw data (top left) shows considerable variation between samples. Data was normalised to the CTCF binding events (top right). Raw ER binding profiles (bottom left) displays different levels in the global binding between each sample; however, no ground truth is known. After normalisation to CTCF PDX01, PDX03 and PDX04 all show similar levels of binding. PDX05 shows reduced genome-wide binding, consistent with being the only PDX to be derived from a tumour with an Allred score of 5 compared to all other samples (Allred = 8).

Ontology	Term Name	Rank	Raw P-Value	FDR Q-Val
MSigDB Perturbation	Genes bound by ESR1 [GeneID=2099] and up-regulated by estradiol [PubChemID=5757] in MCF-7 cells (breast cancer) expressing constitutevly active form of AKT1 [GeneID=207].	1	2.25E-187	7.57E-184
MSigDB Perturbation	Genes bound by ESR1 [GeneID=2099] and up-regulated by estradiol [PubChemID=5757] in MCF-7 cells (breast cancer).	2	2.91E-126	4.90E-123
MSigDB Perturbation	Genes up-regulated in MCF7 cells (breast cancer) at 6 h of estradiol [PubChemID=5757] treatment.	3	6.54E-111	7.33E-108
MSigDB Perturbation	Genes down-regulated in breast cancer tumors (formed by MCF-7 xenografts) resistant to tamoxifen [PubChem=5376].	4	3.47E-106	2.92E-103
MSigDB Perturbation	The 'group 4 set' of genes associated with acquired endocrine therapy resistance in breast tumors expressing ESR1 but not ERBB2 [GeneID=2099;2064].	5	4.40E-93	2.96E-90
MSigDB Perturbation	The 'group 1 set' of genes associated with acquired endocrine therapy resistance in breast tumors expressing ESR1 and ERBB2 [GeneID=2099;2064].	6	1.37E-77	7.70E-75
MSigDB Perturbation	Genes with promoters occupied by PML-RARA fusion [GeneID=5371 and 5914{]} protein in acute promyelocytic leukemia(APL) cells NB4 and two APL primary blasts and based on,Chip-seq data.	8	8.01E-68	3.37E-65
MSigDB Perturbation	Genes up-regulated in luminal-like breast cancer cell lines compared to the basal-like ones.	9	1.28E-67	4.80E-65
MSigDB Perturbation	Genes up-regulated in luminal-like breast cancer cell lines compared to the mesenchymal-like ones.	10	2.40E-63	8.08E-61
MSigDB Perturbation	Genes up-regulated upon overexpression of PARVB [GeneID=29780] in MDA-MB-231 cells (breast cancer) cultured in 3D Matrigel only.	11	3.02E-56	9.23E-54
MSigDB Perturbation	Genes rapidly up-regulated in breast cancer cell cultures by estradiol [PubChem=5757].	12	1.94E-54	5.45E-52
MSigDB Perturbation	Genes up-regulated in MCF7 cells (breast cancer) at 24 h of estradiol [PubChemID=5757] treatment.	13	4.87E-49	1.26E-46
MSigDB Perturbation	Up-regulated genes from the optimal set of 550 markers discriminating breast cancer samples by ESR1 [GeneID=2099] expression: ER(+) vs ER(-) tumors.	14	1.08E-48	2.58E-46
MSigDB Perturbation	Genes down-regulated in MFCF-7 cells (breast cancer) upon stable autocrine expression of HG1 [GeneID=2688].	18	3.09E-41	5.77E-39
MSigDB Perturbation	Genes whose expression negatively correlated with resistance of breast cancer cell lines to dasatinib [PubChem=3062316].	22	1.60E-34	2.45E-32
MSigDB Perturbation	Down-regulated genes in the cancer progenitor (stem) cells corresponding to side population (SP) MCF7 cells (breast cancer) positive for MUC1 [GeneID=4582].	23	2.48E-34	3.63E-32
MSigDB Perturbation	Genes up-regulated in breast cancer samples positive for ESR1 [GeneID=2099] compared to the ESR1 negative tumors.	25	1.55E-33	2.09E-31
MSigDB Perturbation	Genes up-regulated in MCF-7 cells (breast cancer) by estradiol (E2) [PubChem=5757].	27	1.49E-31	1.86E-29
MSigDB Perturbation	Genes within amplicon 16p13 identified in a study of 191 breast tumor samples.	31	1.08E-27	1.17E-25
MSigDB Perturbation	Genes whose expression peaked at 480 min after stimulation of HeLa cells with EGF [GeneID=1950].			

Table S1: **MSigDB Perturbation Terms**. Significantly enriched terms found by GREAT analysis of sites that respond to fulvestrant treatment (FDR=0.01) as established by the ER-CTCF parallel-factor ChIP.

Ontology	Term Name	Rank	Raw P-Value	FDR Q-Val
MSigDB Pathway	Validated nuclear estrogen receptor alpha network	1	2.32E-32	3.07E-29
MSigDB Pathway	FOXA1 transcription factor network	2	1.01E-23	6.70E-21
MSigDB Pathway	C-MYB transcription factor network	3	2.82E-19	1.24E-16
MSigDB Pathway	IGF1 pathway	6	8.59E-18	1.89E-15
MSigDB Pathway	IL2-mediated signaling events	9	1.49E-16	2.19E-14
MSigDB Pathway	SHP2 signaling	10	2.52E-16	3.32E-14
MSigDB Pathway	Glioma	16	1.84E-15	1.52E-13
MSigDB Pathway	IL3-mediated signaling events	17	1.22E-14	9.49E-13
MSigDB Pathway	IL6-mediated signaling events	23	3.79E-14	2.17E-12
MSigDB Pathway	Acute myeloid leukemia	24	7.00E-14	3.85E-12
MSigDB Pathway	Insulin Pathway	32	2.05E-12	8.47E-11
MSigDB Pathway	Mechanism of Gene Regulation by Peroxisome Proliferators via PPARa(alpha)	33	2.59E-12	1.04E-10
MSigDB Pathway	Genes involved in NOTCH1 Intracellular Domain Regulates Transcription	35	3.33E-12	1.26E-10
MSigDB Pathway	Notch-mediated HES/HEY network	40	1.04E-11	3.44E-10
MSigDB Pathway	EPO signaling pathway	45	3.47E-11	1.02E-09
MSigDB Pathway	Validated transcriptional targets of TAp63 isoforms	62	5.73E-10	1.22E-08
MSigDB Pathway	Neuropeptides VIP and PACAP inhibit the apoptosis of activated T cells	94	1.29E-08	1.81E-07
MSigDB Pathway	Keratinocyte Differentiation	102	2.10E-08	2.72E-07
MSigDB Pathway	Stabilization and expansion of the E-cadherin adherens junction	103	2.15E-08	2.76E-07
MSigDB Pathway	Thromboxane A2 receptor signaling			

Table S2: **MSigDB Pathway Terms**. Significantly enriched terms found by GREAT analysis of sites that respond to fulvestrant treatment (FDR=0.01) as established by the ER-CTCF parallel-factor ChIP.

ID	Tissue	Factor	Condition	Rep	Reads	Dup%	ReadL	FragL	RelCC	SSD	RiP%
SLX-8047											
1	MCF7-S2	ER	Fulvestrant	1	345870	4.4	50	103	0.17	3.2	1.4
1	MCF7-S2	ER	none	1	577064	4	50	143	0.86	3.7	3.7
2	MCF7-S2	ER	Fulvestrant	2	914040	4.6	50	157	0.45	5	0.76
2	MCF7-S2	ER	none	2	941137	8.1	50	161	1.1	4.1	4.1
3	MCF7-S2	ER	Fulvestrant	3	836746	5.5	50	109	0.3	4.8	0.42
3	MCF7-S2	ER	none	3	495784	7.6	50	137	0.98	3.1	5.4
4	MCF7-S2	ER	Fulvestrant	4	351296	3.8	50	101	0.0016	4	0.56
4	MCF7-S2	ER	none	4	839376	6.1	50	176	1.2	3.9	3.5
Input	MCF7-S2	Control	Fulvestrant:none	c1	446369	7.5	50	105	-0.017	7.2	0.19
SLX-12998											
1	MCF7-HC11	ER	none	1	1784948	23	50	211	2.3	4.7	17
2	MCF7-HC11	ER	none	2	2056689	25	50	227	2.8	5	23
3	MCF7-HC11	ER	none	3	1824245	23	50	212	2.1	5.4	7.1
4	MCF7-HC11	ER	none	4	1695413	26	50	211	2.4	4.8	14
1	MCF7-HC11	ER	Fulvestrant	1	1814108	23	50	179	0.38	6.2	0.96
2	MCF7-HC11	ER	Fulvestrant	2	1776346	22	50	252	1.7	5.6	4.9
3	MCF7-HC11	ER	Fulvestrant	3	1851604	27	50	191	1.5	3.5	5.3
4	MCF7-HC11	ER	Fulvestrant	4	1651772	23	50	233	0.93	6.2	2.7
Input	MCF7-HC11	Control	Fulvestrant:none	c1	1523785	16	50	103	0.14	5.9	1
SLX14229											
1	MCF7	ERCTCF	Fulvestrant	1	1438445	44	50	102	1.4	4.3	24
1	MCF7	ERCTCF	none	1	2452534	53	50	115	1.6	5	43
2	MCF7	ERCTCF	Fulvestrant	2	4890322	60	50	158	1.9	6.8	45
2	MCF7	ERCTCF	none	2	2965926	54	50	157	1.9	3.7	32
3	MCF7	ERCTCF	Fulvestrant	3	1643976	57	50	156	1.9	4	39
3	MCF7	ERCTCF	none	3	1858857	53	50	102	1.5	4.7	45
Input	MCF7	Control	none:Fulvestrant	c1	494212	44	50	101	0.18	3.5	2.6
SLX14438											
1	MCF7	ERCTCF	none	1	6546303	0*	50	132	1.7	5.8	54
1	MCF7	ERCTCF	Fulvestrant	1	2369926	0*	50	102	1.5	2.5	30
2	MCF7	ERCTCF	none	2	5178114	0*	50	170	2	3.9	42
2	MCF7	ERCTCF	Fulvestrant	2	6064117	0*	50	165	2	5.2	55
3	MCF7	ERCTCF	none	3	3713046	0*	50	107	1.5	4.8	54
3	MCF7	ERCTCF	Fulvestrant	3	3165416	0*	50	166	2.1	3.2	48
Input	MCF7	Control	none:Fulvestrant	c1	1650852	0*	50	101	1.3	0.73	3
SLX-15090											
1	MCF7	H4CTCF	none	1	9023952	26	50	224	2.6	4.5	54
1	MCF7	H4CTCF	Estrogen	1	9443429	26	50	211	2.5	4.7	53
2	MCF7	H4CTCF	none	2	7785593	22	50	216	2.6	3.1	38
2	MCF7	H4CTCF	Estrogen	2	7213665	25	50	212	2.6	3.8	50
3	MCF7	H4CTCF	none	3	6519243	11	50	217	2.5	3.2	44
3	MCF7	H4CTCF	Estrogen	3	7506179	24	50	210	2.5	4	52
Input	MCF7	Control	none:Estrogen	c1	9182077	15	50	199	1.3	0.77	5.8
SLX-15091											
1	MCF7	ERCTCF	none	1	2600080	20	50	165	2.1	2.4	40
1	MCF7	ERCTCF	Estrogen	1	2519064	20	50	166	2.1	2.4	47
2	MCF7	ERCTCF	none	2	2290683	21	50	172	2.2	2.6	50
2	MCF7	ERCTCF	Estrogen	2	1983941	19	50	165	2.1	2.3	50
3	MCF7	ERCTCF	Estrogen	3	2442708	17	50	162	2.4	1.1	16
3	MCF7	ERCTCF	none	3	2041005	20	50	167	2.1	2.5	50
Input	MCF7	Control	none:Estrogen	c1	2446096	11	50	105	1.4	0.6	2.6

ID	Tissue	Factor	Condition	Rep	Reads	Dup%	ReadL	FragL	RelCC	SSD	RiP%
SLX-15439											
PDX01	PDX	ERCTCF	none		2049738	34	50	121	1.9	2.7	39
PDX02	PDX	ERCTCF	none		45300	17	50	107	1.2	0.58	7.9
PDX03	PDX	ERCTCF	none		1543552	29	50	102	1.7	1.3	18
PDX04	PDX	ERCTCF	none		2984111	66	50	103	1.4	1.8	16
PDX05	PDX	ERCTCF	none		4518044	74	50	104	1.6	3.2	23
PDX01	PDX	input	none		1444905	20	50	102	0.4	0.61	
PDX02	PDX	input	none		2888106	20	50	131	0.21	0.64	
PDX03	PDX	input	none		1511232	24	50	135	0.2	0.61	
PDX04	PDX	input	none		1831056	20	50	137	0.1	0.65	
PDX05	PDX	input	none		1731446	21	50	121	0.26	0.61	

Table S3: Summary of ChIPQC results for sequencing librarys. Abbreviations as defined by the ChIPQC package. Sequence data for SLX-14438 was filtered for duplicate reads on merging data from seperate lanes, before ChIP-QC analysis, this is indicated with an asterisk. FastQC analysis of library SLX-14438 gave a duplication rate of 33.21%.
